# QoS-Driven Resource Allocation and Performance Optimization for Aggregated VLC–RF Vehicular Networks

**DOI:** 10.3390/s26144518

**Published:** 2026-07-16

**Authors:** Huanhuan Qin, Xizheng Ke

**Affiliations:** 1 Faculty of Automation and Information Engineering, Xi’an University of Technology, Xi’an 710048, China; xzke@xaut.edu.cn; 2 Shaanxi Civil-Military Integration Key Laboratory of Intelligence Collaborative Networks, Xi’an 710126, China

**Keywords:** aggregated VLC–RF vehicular networks, resource allocation, V2X, QoS, CSI feedback

## Abstract

Visible light communication (VLC) is widely regarded as a key enabler for future vehicular networks, thanks to its extremely large unlicensed bandwidth and non-interference with existing radio frequency (RF) communication networks. With the goal of maximizing the benefits of both RF and VLC technologies, aggregated VLC–RF vehicular networks, in which any vehicle can be served by both RF and VLC access points (APs) concurrently, have recently become a more robust and promising approach for enhancing vehicle-to-everything (V2X) applications and improving the quality-of-service (QoS) of vehicular networks. This paper focuses on the joint spectrum reuse and power allocation problem in aggregated VLC–RF vehicular networks with delayed channel state information (CSI) feedback, where vehicle-to-vehicle (V2V) links opportunistically reuse the RF spectrum allocated to vehicle-to-infrastructure (V2I) links. Specifically, we focus on maximizing the total V2I achievable rate to support high-rate content delivery, and guaranteeing the required reliability of V2V links tasked with exchanging safety-critical information. Furthermore, the sum V2I achievable rate maximization problem is decomposed into four subproblems, which are iteratively solved through an efficient block coordinate descent (BCD)-based alternating optimization algorithm. Moreover, simulation results validate the convergence and efficiency of the proposed algorithm while highlighting the impact of critical parameters on system performance, providing valuable insights for resource allocation in aggregated VLC–RF vehicular networks.

## 1. Introduction

Driven by the explosion in information volume and 6G wireless communication technology, the development of vehicular networks, which aims to improve the daily driving experience and pave the way for intelligent transportation and autonomous vehicles, has attracted significant attention from both industry and academia [1,2]. Thanks to their potential advantages, including reducing traffic fatalities, lowering operational expenditures for fleets, and fostering novel business opportunities [3,4], vehicular networks are undergoing rapid development across numerous countries.

In vehicular networks, the two primary communication types are vehicle-to-infrastructure (V2I) and vehicle-to-vehicle (V2V). The high bandwidth available in V2I communication can support the high data-rate requirements of vehicles, including vehicular entertainment services and dynamic digital map updates. Due to the close physical distance between devices, V2V communication enables the transmission of safety-critical information (including speed, vehicle position, and direction) for autonomous driving and vehicle platooning, which requires stringent reliability and low latency [5]. Long-Term Evolution-Vehicle (LTE-V), a wireless access technology developed for vehicular environments, has been widely adopted owing to its advantages in providing broad coverage and high data rates [6]. To address the limitations of LTE-V’s centralized architecture, the 3rd Generation Partnership Project (3GPP) has adopted device-to-device (D2D) technology to support direct V2V communication [7].

The rapid proliferation of connected vehicles has driven a growing demand for ubiquitous coverage, guaranteed quality-of-service (QoS), and sustainable communication solutions [8]. However, conventional radio-frequency (RF) technologies face challenges in meeting the increasing demands for wireless communication due to their constrained spectrum availability—a critical bottleneck that is expected to intensify with the projected rapid growth in vehicular networks. Visible light communication (VLC) has gained significant interest owing to its inherent advantages, such as ultra-high bandwidth, license-free spectrum availability, and the ability to simultaneously provide illumination and data communication [9]. Research has shown that VLC networks can support data rates in the multi-gigabit-per-second range, making them a highly promising complementary solution to RF networks [10,11]. In addition, VLC offers enhanced security because of the limited penetration capability of visible light signals, as well as reduced power consumption, since the same energy is utilized for both data transmission and illumination [12]. However, the reliance of VLC on line-of-sight (LoS) transmission restricts the coverage area of VLC access points (APs) and increases their sensitivity to LoS blockages, which in turn results in considerable QoS differences among users. Especially for users located at cell edges, where the LoS paths are easily obstructed or where they fall within overlapping zones and suffer inter-cell interference (ICI) from neighboring cells, their QoS can be substantially degraded.

In this scenario, VLC–RF coexisting networks have become promising candidates for future communication systems by exploiting the complementary advantages of both RF and VLC technologies [13]. Specifically, by leveraging the visible light spectrum, VLC helps ease the congestion in the RF spectrum and enables high-speed data transmission, whereas RF provides wide-area coverage and mitigates ICI in VLC networks, thus improving overall system performance [14]. The VLC–RF coexisting networks can be classified into two main types: hybrid networks and aggregated networks [15,16]. In hybrid networks, user association is limited to a single AP type, either RF or VLC, while in aggregated networks, both types of APs are used concurrently to serve each user. Additionally, aggregated VLC–RF networks can provide more reliable communication and greater achievable data rates compared to hybrid VLC–RF networks [16,17]. To improve the performance of integrated VLC–RF networks, a range of resource allocation schemes need to be designed for specific applications and services, taking into account the unique features of both VLC and RF technologies. The key communication performance indicators in the integrated VLC–RF networks include the achievable data rate [15] and energy efficiency (EE) [18,19,20].

On the other hand, the growing demand for wireless vehicular communications is worsening the problem of spectrum shortage in vehicular networks [21,22]. Given that V2I and V2V communications have distinct different QoS requirements while potentially sharing the same spectrum, joint optimization of their resource allocation is essential to achieve green communications with efficient spectrum utilization. In [23], the authors proposed a cross-layer power allocation framework to increase the total throughput of vehicular networks. Their approach employs a multi-tier water-filling algorithm for power distribution and a pricing-based mechanism for relay selection, demonstrating superior performance compared to conventional methods. In [24], the authors investigated a resource allocation problem involving V2I users (IUEs), safety-related V2V users (VUEs), and non-safety VUEs, with the objective of maximizing system throughput. In contrast to separate power allocation schemes, a unified approach coordinating both channel and power resources for vehicle-to-everything (V2X) communications was proposed in [25] using matching theory. In [26], the authors proposed an algorithm that maximizes the rate of IUEs while ensuring that the signal-to-interference-plus-noise ratio (SINR) requirements of VUEs are satisfied. In [27], a resource allocation scheme that jointly considered power control and modulation selection was proposed, taking into account the low-latency and high-reliability demands of LTE-V2V communications. Utilizing Lagrange dual decomposition and stochastic network analysis, the proposed algorithm could effectively identify the optimal solution for the formulated problem. Unlike previous studies that focused on throughput optimization, the study in [28] evaluated system performance using quality of experience (QoE), which exhibits a nonlinear relationship with data rates. To model the QoE saturation effect, a novel utility function was designed as well as a low-complexity heuristic scheme that optimized the overall utility for V2X vehicles. Notably, given the time-varying nature of vehicular communication channels, it is essential to account for rapid channel fluctuations caused by vehicular mobility. In [29], the authors proposed utilizing large-scale fading information while rigorously accounting for small-scale fading effects. In addition, a scenario involving delayed channel state information (CSI) feedback was considered by the authors in [30]. These proposed solutions incorporate both the ergodic capacity properties of IUEs and the reliability requirements for VUE pairs. Although extensive efforts have been devoted to improving the QoS of V2X networks, studies on integrated VLC–RF vehicular networks remain in their early stages. Existing research on aggregated VLC–RF vehicular networks primarily focuses on the data-rate limitations [19] and the EE performance of V2I communications [31]. Building on these findings, we demonstrate that the latent benefits of such integrated networks remain largely unexploited—particularly for advancing QoS-guaranteed V2X communications under adverse transmission conditions. Addressing this research gap is the primary aim of this paper.

To bridge this research gap, we propose a novel framework for D2D-enabled aggregated VLC–RF vehicular networks. The RF AP provides communication services by the RF signals, while the VLC APs transmit data to IUEs using visible light. To ensure eye safety and address other practical limitations of the VLC link [32,33], the VLC network supports only V2I data transmission, while the RF network simultaneously handles both V2I data streams and V2V safety-critical message exchange. To enhance RF spectrum utilization, the downlink spectrum orthogonally assigned to IUEs is reused by VUE pairs [34]. Moreover, to alleviate scheduling complexity and limit interference to IUEs, each VUE pair is restricted from sharing its assigned frequency spectrum with any other VUE pair. Such a setup is crucial for supporting the coexistence of V2I and V2V links within a limited spectrum, but it inevitably requires more advanced interference management mechanisms to address intra-RF cell interference and potential cross-link interference. To address these challenges, resource optimization plays a critical role in V2X communication design, as it enables the development of effective spectrum reuse strategies that meet diverse QoS requirements. These challenges can be alleviated by offloading part of the V2I communication traffic to VLC APs. Because the VLC and RF subnetworks occupy distinct spectrum bands, adding VLC APs helps alleviate the load on the RF spectrum, thereby mitigating intra-cell RF interference. To better highlight the novelty of this work, Table 1 provides a detailed comparison between the proposed framework and closely related studies in terms of channel models, optimized variables, optimization objectives, and solution methods used for discrete resource allocation.

Given the above background, the main contributions of this paper are summarized as follows.

- *System Model and Performance Metrics*: As far as we know, this study is the first to jointly optimize aggregated VLC–RF vehicular networks for both IUEs and VUE pairs, and to propose a comprehensive network model. Specifically, we account for the unavoidable CSI latency resulting from the periodic reporting of vehicular link CSI to the APs in high-mobility vehicular environments. Moreover, our proposed resource allocation problem integrates heterogeneous QoS requirements for IUEs and VUE pairs that align with their supported services, specifically maximizing the total achievable rate for IUEs and ensuring high reliability for VUE pairs.
- *Optimization of Performance and Solution*: Owing to the intricacy of the original optimization problem and the difficulty of solving it directly, we reformulate and decompose it into four subproblems based on the block coordinate descent (BCD) technique: (a) the joint RF transmit power allocation; (b) the spectrum reuse allocation for the VUE pairs; (c) the optical power allocation; and (d) the user allocation. Subsequently, these subproblems are iteratively solved by our proposed alternating iterative optimization algorithm (AIOA), which employs the successive convex approximation (SCA) method, the relaxed iterative method, and the minorization–maximization (MM) algorithm for the corresponding subproblems, respectively.
- *Resource Allocation and Performance Verification*: This paper provides a theoretical analysis of the computational complexity of the proposed algorithm. Moreover, simulation results verify that the aggregated VLC–RF networks achieve higher V2I data rates by applying the proposed methods than other baselines while satisfying the diverse QoS requirements of V2X communications and maintaining acceptable computational complexity. The simulation results further investigate the influence of key parameters on the sum V2I achievable rate in aggregated VLC–RF vehicular networks, demonstrating that carefully designed parameter configurations can further enhance the sum V2I achievable rate.

This paper is structured as follows. Section 2 describes the channel models of both the VLC and RF subnetworks, followed by the formulation of the optimization problem. Section 3 proposes an effective algorithm and the corresponding approaches for the four subproblems, along with an analysis of its convergence and computational complexity. The simulation results and conclusions are presented in Section 4 and Section 5, respectively.

*Notation*: Scalars and matrices are denoted by *a* and **A**, respectively. A represents the defined set, and arg max (·) denotes the argument that maximizes the corresponding expression. Moreover, ‖·‖*_F_*, ∂, (·)*^T^*, and |·| stand for the Frobenius norm, the gradient operator, the transpose, and the absolute value, respectively.

## 2. System Model

**Figure 1:**
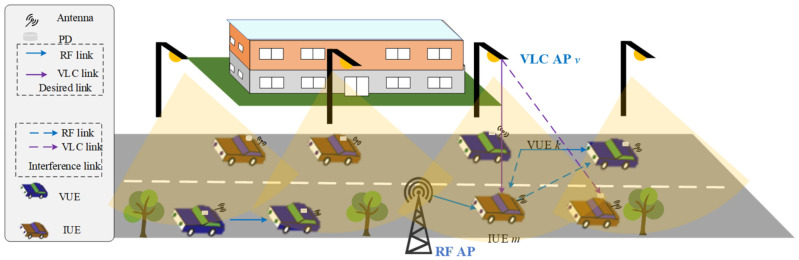
System model of the proposed D2D-enabled aggregated VLC–RF vehicular networks.

This paper studied the aggregated VLC–RF vehicular networks depicted in Figure 1, in which *V* VLC APs are uniformly deployed along a multi-lane urban road, and one RF AP is installed in the central area to support V2I communications. The set of VLC APs is represented by V = {1, 2, …, *V*}. These APs are linked to the centralized control unit for global management. As two essential components of vehicular networks, V2I and V2V communications are employed to support a wide range of vehicular applications. In future transportation scenarios, an increasing number of traffic-oriented and entertainment services, including video streaming and crowdsensing, will rely on high-capacity V2I communications. In parallel, safety-critical data must be transmitted to nearby vehicles reliably and timely through V2V links [35]. In this model, we assume that there are *M* IUEs that require high-capacity services, and *K* VUE pairs carry out local data exchange through D2D communication. The set of all IUEs is defined as M = {1, 2, …, *M*}, while the set of all VUE pairs is represented as K = {1, 2, …, *K*}. Without loss of generality, the number of VUE pairs is greater than that of IUEs, i.e., *K* ≥ *M*. The spectrum bands exploited by IUEs are reused by VUE pairs to improve the spectrum efficiency. Furthermore, all vehicles move at random speeds and are equipped with a photo-detector (PD) and an antenna, allowing them to be simultaneously served by both a VLC AP and the RF AP. Additionally, it is assumed that all APs have accurate knowledge of the CSI for the links directly connected to them, since these can be locally estimated. In contrast, the CSI of vehicular links is obtained through periodic feedback with an interval of *T*, which results in feedback delay.

### 2.1. The VLC Subnetwork

Without loss of generality, VLC mainly relies on the LoS channel, while the non-line-of-sight (NLoS) channel can be neglected owing to the weakness of diffuse reflection [19,33]. The LoS link probability *ρ* between a VLC AP and an IUE is assumed to follow a uniform distribution. When only LoS paths are taken into account, the VLC channel gain from VLC AP *v* to IUE *m* can be modeled using the Lambertian model, which can be formulated as [36]:

(1) $$h_{v,m}=\frac{\rho (\Omega +1)\varepsilon_{m}\varpi_{m}}{2\pi {\left(d_{v,m}\right)}^{2}}\cos^{\Omega }\left(\varphi_{v,m}\right)\cos\left(\xi_{v,m}\right)G\left(\xi_{v,m}\right)T_{s}\left(\xi_{v,m}\right),$$

where Ω = –log_2_(cos(*φ*_1/2_))^–1^ is the order of the Lambertian emission with *φ*_1/2_ being the LED’s semi-angle at half power; *ε_m_* and *ϖ_m_* are the detection area of the PD and responsivity of the *m*-th IUE, respectively; *d_v_*_,*m*_, *φ_v_*_,*m*_, and *ξ_v_._m_* are the distance, the irradiance angle and the angle of incidence between the AP *v* and IUE *m*, respectively; *T_s_*(*ξ_v_*_,*m*_) is the optical filter gain at IUE *m*; and the gain of the optical concentrator at the IUE *m*, denoted as *G*(*ξ_v_._m_*), can be given by:

(2) $$G\left(\xi_{v,m}\right)=\left\{\begin{array}{ll} \frac{\chi^{2}}{\sin^{2}\xi_{\mathrm{FOV}}}, & \;\mathrm{if}\;\xi_{v,m}\leq \xi_{\mathrm{FOV}},\\ 0, & \;\mathrm{if}\;\xi_{v,m}>\xi_{\mathrm{FOV}}, \end{array}\right.$$

where *ξ*_FOV_ and *χ* are the field of view (FOV) and the refractive index of the PD, respectively. Since the CSI of the links directly connected to the VLC APs can be locally estimated at the VLC APs, the CSI in the VLC network is assumed to be accurately known.

For the VLC subnetwork, each VLC AP can serve multiple IUEs simultaneously, with its baseband modulation bandwidth denoted as *Q*_1_. In addition, the power allocation matrix for the IUEs is denoted by $P=\left\{p_{v,m},\forall v,m\right\},$ where *p_v_*_,*m*_ denotes the transmit power from VLC AP *v* to IUE *m*. To characterize the association between VLC APs and IUEs, a binary matrix $A=\left\{\alpha_{v,m},\forall v,m\right\}$ is defined, which is given as follows:

(3) $$\alpha_{v,m}=\left\{\begin{array}{ll} 1, & if\;\mathrm{IUE}\;m\;is\;served\;by\;\mathrm{VLC}\;\mathrm{AP}\;v,\\ 0, & otherwise. \end{array}\right.$$

Then, the optical interference received by IUE *m* originates from the LoS channels of other VLC APs. Hence, the SINR between VLC AP *v* and IUE *m* is expressed as

(4) $$\gamma_{v,m}=\frac{\alpha_{v,m}p_{v,m}h_{v,m}^{2}}{\sum_{v’=1,v’\neq v}^{V}\alpha_{v’,m}p_{v’,m}h_{v’,m}^{2}+\sigma_{\mathrm{VLC}}^{2}},$$

where $\sigma_{\mathrm{VLC}}^{2}$ denotes the variance of the noise in the VLC network. For IUE *m* associated with the *v*-th VLC AP, the interference caused by other VLC APs *v*′ ≠ *v* corresponds to the first denominator term in Equation (4). Then, the corresponding achievable data rate of IUE *m* from VLC AP *v* can be expressed as [36]:

(5) $$R_{v,m}=\frac{Q_{1}}{2M}\log_{2}\left(1+\frac{e}{2\pi }\gamma_{v,m}\right).$$

Following that, the total achievable data rate received by IUE *m* in the VLC subnetwork is expressed as:

(6) $$R_{m}=\sum_{v=1}^{V}R_{v,m}.$$

### 2.2. The RF Subnetwork

For the RF subnetwork, due to the fast time-varying nature of V2X channels, instantaneous CSI is often difficult to obtain accurately and in a timely manner. Therefore, this paper exploits slowly varying large-scale CSI to satisfy the QoS requirements of both V2I and V2V links. Thus, the channel gain between IUE *m* and the RF AP can be expressed as [37]:

(7) $$h_{b,m}={\left|\zeta_{b,m}\right|}^{2}\lambda_{b,m}\kappa d_{b,m}^{-\beta },$$

where *ζ_b_*_,*m*_ is the small-scale fast fading component of the link between the *m*-th IUE and RF AP, which follows complex Gaussian distribution with zero mean and unit variance; *λ_b_*_,*m*_ is the log-normal shadowing random variable with standard deviation *ν* for the link between the *m*-th IUE and RF AP; κ is the path loss at the reference distance *d*_0_ = 1 m; *d_b_*_,*m*_ denotes the distance between the *m*-th IUE and the RF AP; and *β* is the corresponding path loss exponent. In the vehicular network channel model adopted in this paper, since V2I links typically have longer propagation distances and are more susceptible to blockage caused by buildings, roadside infrastructure, and large vehicles, their large-scale channel fluctuations are generally more significant than those of short-distance V2V links. Therefore, a larger shadow fading standard deviation *ν* is adopted for V2I links. For the V2I and V2V links, different values of κ and *β* are adopted, and the Doppler effect caused by vehicle mobility is assumed to be perfectly compensated.

Since the CSI of the links directly associated with the RF AP can be accurately estimated at the AP side, this type of CSI is assumed to be perfectly known. In contrast, the CSI of V2V links needs to be periodically fed back to the RF AP according to the feedback period *T*, and thus inevitably suffers from a certain feedback delay. To characterize the time-varying nature of the V2V channel under such delay conditions, this paper introduces a first-order Gauss–Markov process [30] to model the fast-fading variation within the feedback period *T*, which is expressed as follows:

(8) $$h=\epsilon \hat{h}+e$$

where $\hat{h}$ and *h* are the channels in the previous and current time, respectively; *e* is the channel discrepancy term distributed according to $CN\left(0,1-\epsilon^{2}\right)$ and independent of $\hat{h}$; coefficient *ϵ* quantifies channel correlation between the two consecutive time slots. For the Jakes’ model [30], *ϵ* is given by:

(9) $$\epsilon =J_{0}\left(2\pi f_{d}T\right)$$

where *J*_0_(⋅) is the zero-order Bessel function of the first kind and *f_d_* = *c_v_f*_c_/*c* is the maximum Doppler frequency with *c* = 3 × 10^8^ m/s, *c_v_* being the vehicle speed, and *f*_c_ being the carrier frequency. In addition, the RF AP can serve multiple IUEs simultaneously, and its total bandwidth is denoted by *Q*_2_. We define $p_{m}^{c}$ and $p_{k}^{d}$ as the transmit powers of the *m*-th IUE and the *k*-th VUE pair’s transmitter, respectively. Building upon this, the RF spectrum reuse problem addressed involves the joint optimization of the power allocation $D=\left\{p_{m}^{c},p_{k}^{d},\forall m,k\right\}$ and the spectrum reuse strategy for the VUE pairs $B=\left\{b_{m,k},\forall m,k\right\}$. To this end, the SINR from the RF AP to IUE *m* can be expressed as:

(10) $$\gamma_{m}=\frac{p_{m}^{c}h_{b,m}^{2}}{\sum_{k\in K}b_{m,k}p_{k}^{d}h_{m,k}^{2}+\sigma_{\mathrm{RF}}^{2}},$$

where *h_m_*_,*k*_ is the channel gain between IUE m and the *k*-th VUE pair’s transmitter; $\sigma_{\mathrm{RF}}^{2}$ denotes the variance of the noise in the RF network. Subsequently, the corresponding achievable data rate for IUE *m* can be written as:

(11) $$S_{m}=\frac{Q_{2}}{2M}\log_{2}\left(1+\gamma_{m}\right).$$

For V2V links, the SINR received by the k-th VUE pair can be expressed as:

(12) $$\gamma_{k,l}=\frac{p_{k,l}h_{k}^{2}}{\sum_{m\in M}b_{k,l}p_{m,l}h_{b,k}^{2}+\sigma_{\mathrm{RF}}^{2}}=\frac{p_{k,l}\left(\epsilon_{k}^{2}{\left|{\hat{h}}_{k}\right|}^{2}+{\left|e_{k}\right|}^{2}\right)}{\sum_{m\in M}b_{k,l}p_{m,l}\left(\epsilon_{b,k}^{2}{\left|{\hat{h}}_{b,k}\right|}^{2}+{\left|e_{b,k}\right|}^{2}\right)+\sigma_{\mathrm{RF}}^{2}},$$

where *h_k_* denotes the channel gain for *k*-th VUE pair and *h_b_*_,*k*_ represents the channel gain from the RF AP to the receiver of *k*-th VUE pair.

### 2.3. Optimization Problem Formulation

For the aggregated VLC–RF vehicular networks, V2I links are intended to provide high-rate infotainment applications for mobile IUEs, whereas V2V links are dedicated to ensuring the reliable delivery of safety-critical information for VUE pairs. Therefore, our objective is to maximize the sum achievable rate of the *M* IUEs, given the total power constraint and the minimum data-rate requirements for the IUEs, while guaranteeing that all VUE pairs satisfy their minimum SINR requirements [6,34,38]. Accordingly, the V2I achievable data rate can be expressed as:

(13) $$\Gamma =\sum_{m=1}^{M}\left(R_{m}+S_{m}\right).$$

Additionally, the overall power consumption is calculated as:

(14) $$P_{T}=VU_{1}+U_{2}+\sum_{v=1}^{V}\sum_{m=1}^{M}p_{v,m}++\sum_{m=1}^{M}p_{m}^{c}$$

where *U*_1_ and *U*_2_ represent the static optical power consumption for VLC AP illumination and the static circuit power consumed by the RF AP, respectively [31]. Consequently, the problem of maximizing the V2I achievable rate for aggregated VLC–RF vehicular networks by jointly optimizing the user allocation **A**, the spectrum reuse design **B** for V2V links, the joint RF transmit power allocation **D**, and the optical power allocation **P** can be formulated as:

(15) $$(P0)\underset{\begin{array}{l} \left\{A,P,B,D\right\} \end{array}}{\max}\Gamma ,$$

(15a) $$s.t.\;p_{v,m}\geq 0,\;\forall v\in V,\forall m\in M,$$

(15b) $$\sum_{m=1}^{M}p_{v,m}\leq \min P_{v,\max}$$

(15c) $$p_{m}^{c}\geq 0,p_{k}^{d}\geq 0,\;\forall m\in M,\forall k\in K,$$

(15d) $$\sum_{m=1}^{M}p_{m}^{c}\leq W_{\max},$$

(15e) $$VU_{1}+U_{2}+\sum_{v=1}^{V}\sum_{m=1}^{M}p_{v,m}+\sum_{m=1}^{M}p_{m}^{c}\leq U_{T}$$

(15f) $$0\leq p_{k}^{d}\leq P_{\max}^{d},\;\forall k\in K,$$

(15g) $$R_{m}+S_{m}\geq R_{\min},\;\forall m\in M,$$

(15h) $$\alpha_{v,m}\in \{0,1\},\;\forall v\in V,\forall m\in M,$$

(15i) $$\sum_{m=1}^{M}\alpha_{v,m}\leq 1,\;\forall v\in V,$$

(15j) $$b_{m,k}\in \{0,1\},\;\forall m\in M,\forall k\in K,$$

(15k) $$\sum_{m=1}^{M}b_{m,k}\leq 1,\;\forall k\in K,$$

(15l) $$\sum_{k=1}^{K}b_{m,k}\leq 1,\forall m\in M,$$

(15m) $$\gamma_{k}\geq \gamma_{\mathrm{th}},\forall k\in K.$$

The constraints of problem (P0) are detailed as follows.

**Transmit Power Constraints:** Constraints (15a) and (15c) guarantee non-negative power allocation for the VLC and RF subnetworks, respectively. Both the VLC APs and the RF AP are required to operate within their individual maximum transmit power constraints, as enforced by constraints (15b) and (15d), where *P_v_*_,max_ and *W*_max_ denote the maximum allowable power for VLC AP *v* and the RF AP, respectively. To ensure that the total power consumption of the aggregated vehicular network does not exceed the threshold *U*_T_, the corresponding power constraint is given by (15e). Constraint (15f) defines the transmit power range of VUE pair *k*, where $P_{max}^{d}$ is the maximum allowable power for each VUE pair.

**Date Rate Constraints:** To ensure that the QoS requirements of all IUEs are satisfied, the achievable data rate of each IUE must exceed the minimum threshold *R*_min_, as specified in constraint (15g).

**User Association Constraints:** Constraints (15h) and (15i) ensure that all its elements are binary, and that each IUE is allocated to at most one VLC AP.

**RF Spectrum Reuse Allocation Constraints:** Constraint (15j) ensures that its elements are binary. Constraints (15k) and (15l) define the spectrum reuse mechanism for V2X communications, meaning that the spectrum assigned to a single IUE can be reused by at most one VUE pair, and likewise, each VUE pair can reuse the spectrum allocated to only one IUE.

**Reliability requirements:** Owing to the strict reliability requirements imposed on VUE pairs, the SINR of each VUE pair must exceed the minimum threshold *γ*_th_ as specified in (15m).

## 3. Proposed Resource Allocation Algorithm

The problem (P0) falls into the class of mixed-integer nonlinear programming (MINLP) problems, which is typically computationally challenging and has been proven to be NP-hard [39]. In problem (P0), the optimization is conducted over four interdependent variable sets: the user allocation **A**, the spectrum reuse design **B** for V2V links, the joint RF transmit power allocation **D**, and the optical power allocation **P**. Driven by the broad applicability and high efficiency of the BCD algorithm, we alternately optimize **A**, **B**, **P**, and **D** while keeping the remaining variables fixed, which effectively handles the coupling among the optimization variables. This leads to an alternating iterative algorithm for obtaining an optimal approximate solution.

### 3.1. Joint Optimization of RF Transmit Power Allocation

Given fixed **A, B**, and **P**, the achievable data rate *R_m_* is constant. Thus, the problem (P0) can be expressed as:

(16) $$\begin{array}{l} \;(P1)\;\underset{D}{\max}\sum_{m=1}^{M}S_{m}\left(D\right)\\ \;s.t.\;(15c)\text{–}(15f),(15m), \end{array}$$

(16a) $$S_{m}\geq R_{\min}-R_{m},\forall m\in M.$$

Since the objective function of problem (P1) is a sum of achievable rates involving fractional SINR expressions, and constraint (15m) is also non-convex, problem (P1) is a non-convex fractional programming problem and is difficult to solve directly. In this section, the quadratic transform (QT) method is first adopted to equivalently transform the fractional terms in the above objective function, thereby reformulating it into the difference of two concave functions [40]. Specifically, by introducing a slack vector $\Theta =\left[\Theta_{1},\Theta_{2},\cdots ,\Theta_{M}\right]$ consisting of *M* complex-valued elements, which is also referred to as the quadratic parameter, the SINR term in (10) can be further expressed as follows:

(17) γm,l=2ReΘmHpm,l1/2hb,m−ΘmH∑k∈Kbk,lpk,lhm,k2+σRF2Θm

When the RF transmit power allocation matrix **D** is given, the optimal solution of the quadratic parameter $\Theta_{m}$ can be obtained by differentiating the corresponding function, i.e., by setting:

(18) $$\frac{\partial \gamma_{m}\left(\Theta_{m},D\right)}{\partial \Theta_{m}}=0$$

When the above condition is satisfied, the optimal solution corresponding to (18) can be written as:

(19) $$\Theta_{m}^{*}=\left[{\left(p_{m,l}\right)}^{1/2}h_{b,m}\right]{\left(\sum_{k\in K}b_{k,l}p_{k,l}h_{m,k}^{2}+\sigma_{\mathrm{RF}}^{2}\right)}^{-1}$$

When the quadratic parameter $\Theta_{m}=\Theta_{m}^{*}$ corresponding to each IUE attains its optimal value, the QT-transformed expression in (17) is equivalent to the original fractional SINR expression in (10). By introducing the QT method, the original problem (P1) can be reformulated as follows:

(20)  (P1-1) maxD,ΘQ22∑m=1M∑l=1Llog21+2ReΘmHpm,l1/2hb,m−ΘmH∑k∈Kbk,lpk,l1/2hm,k+σRF2Θm

(20a) s.t. (15c)–(15f),(15m),

(20b) $$S_{m}\geq R_{\min}-R_{m},\forall m\in M$$

When the quadratic parameter $\Theta_{m}$ is fixed, the constraint is (15c)–(15f) convex, and the minimum channel capacity constraint (20b) is also convex, whereas the SINR threshold constraint (15m) for VUE pairs is non-convex. Therefore, subproblem (P1-1) remains non-convex. To transform the constraint into a convex one, it is represented by the following second-order cone constraint:

(21) 1γthpm,l1/2hb,m≥∑k∈Kbk,lpk,lhm,k2+σRF212,Impm,l1/2hb,m=0

To handle the non-convexity of the objective function in (20), we introduce two auxiliary slack variables, auxiliary slack variables $\Phi =\left[\Phi_{1},\Phi_{2},\ldots ,\Phi_{M}\right]$ and $\lambda =\left[\lambda_{1},\lambda_{2},\ldots ,\lambda_{M}\right]$, both of which contain *M* real-valued elements. After introducing the above variables, the following inequalities can be obtained

(22) $$\Phi_{m}\geq \sum_{k\in K}b_{k,l}p_{k,l}h_{m,k}^{2}+\sigma_{\mathrm{RF}}^{2}$$

(23) λm≤2ReΘmHpm,l1/2hb,m−ΘmH∑k∈Kbk,lpk,lhm,k2+σRF2Θm

According to (22), (23) can be reformulated as:

(24) λm≤2ReΘmHpm,l1/2hb,m−ΘmHΦmΘm

Therefore, by constructing a concave approximation of the objective function (20) of subproblem (P1-1), problem (P1-1) can be reformulated as the following convex optimization problem:

(25) $$\begin{array}{c} (P1\text{-}2)\;\underset{D,\Phi ,\lambda }{\max}\;\frac{Q_{2}}{2}\sum_{m\in M}\log_{2}(1+\lambda_{m})\\ \;s.t.\;(15c)\text{–}(15f),(21),(22),(24), \end{array}$$

In subproblem (P1-2), a new objective function (25) and the inequality constraints (21), (22), and (24) are introduced. The new objective function (25) and the inequality constraints (22) and (24) are obtained by replacing the original objective function (20) in problem (P1-1). That is, the new objective function (25), together with the inequality constraints (22) and (24), is equivalent to the original objective function (20) in (P1-1). The inequality constraint (21) is used to replace constraint (15m) in optimization problem (P1-1). By introducing the new constraint (22), the objective function of the optimization subproblem (P1-2) can serve as a lower bound for the objective function of the optimization subproblem (P1-1). Moreover, constraints (21) and (24) are both convex. Hence, optimization subproblem (P1-2) has been transformed into a standard convex optimization problem, which can be efficiently solved by standard convex optimization solvers such as CVX [41]. Therefore, the optimal solution of problem (P1-2) can be regarded as an approximately optimal solution to problem (P1).

### 3.2. Optimization of RF Spectrum Reuse Allocation

When **P**, **A**, and **D** are fixed, the achievable data rate *R*_m_ remains constant, which enables problem (P0) to be simplified as:

(26) $$\begin{array}{c} \;(P2)\;\underset{B}{\max}\sum_{m=1}^{M}S_{m}(B)\\ \;s.t.\;(15d)\text{–}(15g),(15j)\text{–}(15m). \end{array}$$

Owing to the non-convexity of the objective function and the binary nature of the decision variables **B**, problem (P2) constitutes a mixed-integer non-convex optimization problem. To improve the tractability of the optimization problem presented in (26), we first relax the binary variables in (15j) to continuous variables, thereby obtaining the reformulated problem:

(27) $$(P2\text{-}1)\;\underset{B}{\min}\;-\sum_{m=1}^{M}S_{m}(B)$$

(27a) $$s.t.\;0\leq b_{m,k}\leq 1,\forall m\in M,\forall k\in K,$$

(27b)  (15c)–(15g),(15k)–(15m).

To improve the tractability of problem (27), we first define auxiliary slack variables $\omega =\left\{\omega_{m}=\log_{2}\left(1+\gamma_{m}\right),\forall m\right\}$. Subsequently, the binary constraints in (27a) are relaxed to allow continuous values, which introduces the bilinear product *b_m_*_,*k*_*ω_m_* into the objective function. At the same time, the constraint $\omega_{m}\leq \log_{2}\left(1+\gamma_{m}\right)$ must hold. To address these non-convex terms, −*b_m_*_,*k*_*ω_m_* is rewritten as the difference of two convex functions, which can be expressed as:

(28) $$\begin{array}{ll} -\omega_{m}b_{m,k} & =\frac{1}{2}b_{m,k}^{2}+\frac{1}{2}\omega_{m}^{2}-\frac{1}{2}{\left(\omega_{m+}b_{m,k}\right)}^{2}\\ & \overset{\left(a\right)}{\leq }\frac{1}{2}b_{m,k}^{2}+\frac{1}{2}\omega_{m}^{2}-\frac{1}{2}{\left(b_{m,k}^{\left(t\right)}+\omega_{m}^{\left(t\right)}\right)}^{2}\\ & -\left(b_{m,k}-b_{m,k}^{\left(t\right)}\right)\left(b_{m,k}^{\left(t\right)}+\omega_{m}^{\left(t\right)}\right)-\left(\omega_{m}-\omega_{m}^{\left(t\right)}\right)\left(b_{m,k}^{\left(t\right)}+\omega_{m}^{\left(t\right)}\right)\\ & ={ℵ}_{m}^{\mathrm{UB}}, \end{array}$$

where (*a*) is obtained from the well-known fact that the first-order Taylor expansion of a concave function provides a global upper bound on the function at all points [34], where $b_{m,k}^{\left(t\right)}$ and $\omega_{m}^{\left(t\right)}$ represent the reference values in the *t*-th iteration. In a similar manner, since $\log_{2}\left(1+\gamma_{m}\right)$ is convex, it can be lower-bounded by its first-order Taylor expansion evaluated at reference point $\omega_{m}^{\left(t\right)}$, which is given by:

(29) $$\log_{2}\left(1+\gamma_{m}\right)\geq \log_{2}\left(1+\gamma_{m}^{\left(t\right)}\right)+\sum_{k\in K}\delta_{m,k}\left(b_{m,k}-b_{m,k}^{\left(t\right)}\right)=\Phi_{m}^{\mathrm{LB}},$$

where *δ_m_*_,*k*_ denotes a derivative-associated coefficient with negligible influence and is therefore omitted. Consequently, problem (P2-1) is further reformulated as:

(30) $$(P2\text{-}2)\;\underset{B,\omega }{\min}\frac{Q_{2}}{2}\sum_{m\in M}{ℵ}_{m}^{\mathrm{UB}}$$

(30a) $$s.t.\;b_{m,k}\in \left[0,1\right],\;\forall m\in M,\forall k\in K,$$

(30b) $$\omega_{m}\leq \Phi_{m}^{\mathrm{LB}},\forall m\in M,$$

(30c) $$S_{m}\geq R_{\min}-R_{m},\;\forall m\in M,$$

(30d) (15c)–(15f),(15k)–(15m).

Since problem (P2-2) is convex, its optimal solution can be efficiently computed using standard convex solvers such as CVX [41]. Since optimization problem (P2-2) is equivalent to optimization problem (P2-1), the optimal solution of problem (P2-2) can also satisfy optimization problem (P2-1), and is also the optimal solution of optimization problem (P2-1). Based on the lower-bound approximation constraint (34b), it can be derived that the optimal value of optimization problem (P2-1) serves as a lower bound for optimization problem (P2). Therefore, the optimal feasible solution of optimization problem (P2-1) is also an approximately optimal solution to problem (P2). The method described above for solving subproblem (P2) is called the relaxed iterative method.

*Remark 1:* The RF spectrum variables **B** derived from problem (P2-2) are relaxed to continuous values in the range [0,1]. Therefore, a reconstruction procedure is required to recover the corresponding binary allocation variables. To reconstruct the binary variables, the rule is to maximize the objective function of subproblem (P2). Accordingly, the optimal indices are given by:

(31) $$m^{*}=\arg\max_{m}\frac{\partial \left(\sum_{m\in M}S_{m}\right)}{\partial b_{m,k}},$$

(32) $$\;k^{*}=\arg\max_{k}\frac{\partial \left(\sum_{m\in M}S_{m}\right)}{\partial b_{m,k}},$$

where $b_{m^{*},k}^{*}=1$ and $b_{m,k^{*}}^{*}=1$ represent the suboptimal RF spectrum allocation variables, respectively.

### 3.3. Optimization of Optical Power Allocation

Given the fixed **A**, **B**, and **D**, the RF achievable data rate *S_m_* is constant. Thus, the problem (P0) can be written as:

(33) $$\begin{array}{c} (P3)\;\underset{P}{\max}\sum_{m=1}^{M}R_{m}\left(P\right)\\ \;s.t.\;(15a),(15b),(15e), \end{array}$$

(33a) $$R_{m}\geq R_{\min}-S_{m},\forall m\in M.$$

To address the non-convex subproblem (P3), the MM algorithm is employed, which is a representative technique within the SCA framework. The MM algorithm has been widely applied in signal processing, communications, networking, and machine learning [42]. By iteratively solving a sequence of convex surrogate subproblems, the algorithm is ensured to converge to a KKT point of the original non-convex optimization problem [40]. First, we define the achievable data rate as $F(\varsigma )=Q_{1}\log_{2}(1+\varsigma )/2$, where *ς* denotes the SINR of the IUEs in the VLC subnetwork. Let *ς*_0_ denote an arbitrary non-negative constant, a new function $F_{1}(\varsigma ,\varsigma_{0})$ is formulated as:

(34) $$F_{1}(\varsigma ,\varsigma_{0})=Q_{1}\left[\vartheta \left(\varsigma_{0}\right)\log_{2}(\varsigma )+\varepsilon \left(\varsigma_{0}\right)\right]$$

where *ϑ* and *ε* are two functions of *ς*_0_, and they are given as:

(35) $$\vartheta (\varsigma_{0})=\frac{\varsigma_{0}}{2\left(1+\varsigma_{0}\right)},$$

(36) $$\varepsilon \left(\varsigma_{0}\right)=\frac{1}{2}\log_{2}\left(1+\varsigma_{0}\right)-\frac{\varsigma_{0}}{2\left(1+\varsigma_{0}\right)}\log_{2}\varsigma_{0},$$

Generally, the new function satisfies three properties. First, $F(\varsigma )\geq F_{1}(\varsigma ,\varsigma_{0})$ is always held for any positive real number *ς*. Second, $F(\varsigma_{0})=F_{1}(\varsigma_{0};\varsigma_{0})$ at the point *ς*_0_ [43]. Finally, the two functions F(ς) and $F_{1}(\varsigma ,\varsigma_{0})$ have equal tangent slopes at *ς* = *ς*_0_, which can be expressed as:

(37) $${\left.\frac{\partial F(\varsigma )}{\partial \varsigma }\right|}_{\varsigma =\varsigma_{0}}={\left.\frac{\partial F_{1}\left(\varsigma ;\varsigma_{0}\right)}{\partial \varsigma }\right|}_{\varsigma =\varsigma_{0}}.$$

In summary, function $F_{1}(\varsigma ,\varsigma_{0})$ serves as a lower bound for function F(ς), and they share the same derivative and value at point *ς* = *ς*_0_ [31]. Consequently, the lower bound function for the target achievable data rate function $R_{v,m}(P)=F\left(\gamma_{v,m}(P)\right)$ can be given by $F_{1}(\gamma_{v,m}(P);\varsigma_{0})$, where the constant parameter $\varsigma_{0}=\gamma_{v,m}(P^{\left(t\right)})$ represents the tangential point in the *t*-th iteration. Therefore, the approximate function can be expressed as:

(38) $${\underline{R}}_{m}\left(P;P^{\left(t\right)}\right)=Q_{1}\left[\vartheta \left(\varsigma_{0}\right)\log_{2}\left(\frac{e}{2\pi }\gamma_{v,m}(P)\right)+\varepsilon \left(\varsigma_{0}\right)\right]\;,$$

where the constant parameter $\varsigma_{0}=\frac{e}{2\pi }\gamma_{v,m}\left(P^{\left(t\right)}\right)$ is the tangent point in the *t*-th iteration. Although a lower bound of the original objective has been derived, *R_m_* remains non-convex because the logarithmic term still contains a fractional structure. To address this issue, an approximation is defined as:

(39) $$F_{2}\left(\varsigma ;\varsigma_{1}\right)=\log_{2}\left(\varsigma_{1}\right)+\left(\varsigma /\varsigma_{1}-1\right)/\ln2$$

which represents the first-order Taylor linearization of $\log_{2}\left(\varsigma \right)$. Accordingly, *R_m_* can be relaxed as:

(40) $$\begin{array}{ll} {\underline{\underline{R}}}_{m}\left(P;P^{\left(t\right)}\right) & =Q_{1}\left[\vartheta \left(\varsigma_{0}\right)\log_{2}\left(p_{v,m}h_{v,m}^{2}\right)+\varepsilon \left(\varsigma_{0}\right)\right]\\ & -Q_{1}\vartheta \left(\varsigma_{0}\right)F_{2}\left(\sum_{v’=1,v’\neq v}^{V}p_{v’,m}h_{v’,m}^{2}+\sigma_{\mathrm{VLC}}^{2};\varsigma_{1}\right), \end{array}$$

where *ς*_1_ is a constant parameter computed based on **P**^(*t*)^, which can be expressed as:

(41) $$\varsigma_{1}=\sum_{v’=1,v’\neq v}^{V}p_{v’,m}^{\left(t\right)}h_{v’,m}^{2}+\sigma_{\mathrm{VLC}}^{2}.$$

Based on the first-order Taylor expansion and the properties of the MM algorithm, a partial order relation can be established as:

(42) $$R_{m}(P)\geq {\underline{R}}_{m}\left(P;P^{\left(t\right)}\right)\geq {\underline{\underline{R}}}_{m}\left(P;P^{\left(t\right)}\right).$$

For m∈M, the function ${\underline{\underline{R}}}_{m}\left(P;P^{\left(t\right)}\right)$ is concave [44]. Consequently, the lower bound function of ${\underline{\underline{R}}}_{m}\left(P;P^{\left(t\right)}\right)$ serves as the tractable objective for iterative optimization, and the original subproblem (P3) is reformulated as:

(43) $$(P3\text{-}1):\underset{P}{\max}\sum_{v=1}^{V}\sum_{m=1}^{M}{\underline{\underline{R}}}_{v,m}\left(P;P^{\left(t\right)}\right)$$

(43a) s.t. (15a),(15b),(15e),

(43b) $$\sum_{m=1}^{M}{\underline{\underline{R}}}_{m}\left(P;P^{\left(t\right)}\right)\geq R_{\min}-S_{m},\forall m\in M.$$

Since the objective function is composed of multiple concave terms and constraints (43a) and (43b) are convex, problem (P3-1) constitutes a convex optimization problem. Therefore, the optimal optical transmit power allocation for problem (P3-1) can be efficiently obtained using interior-point methods (IPMs) [41]. The procedure for solving problem (P3-1) is outlined in Algorithm 1. At each iteration, an IPM is first applied to solve (P3-1), yielding the matrix P^(*t*)^. Using the obtained solution, the parameters *ς*_0_ and *ς*_1_ for each IUE are subsequently recalculated and updated. The process repeats until the convergence criterion is met, thus the final optimal optical power allocation matrix P^*^ is determined. Therefore, the optimal solution of optimization problem (P3-1) also satisfies all the constraints of optimization problem (P3), and thus the optimal solution of problem (P3-1) can be regarded as an approximately optimal solution to problem (P3).
**Algorithm 1.** MM-based Optical Power Allocation Algorithm1: **Input:** The convergence threshold *ϵ_1_>* 0, and $\left\{h_{v,m}\},\{h_{b,m}\},\{h_{m,k}\},\{h_{k}\},\{h_{b,k}\right\}$.2: **Initialization:** iteration rounds *t* = 0, *m* = 1, a feasible solution **P**^(0)^, and initialize *ς*_0_ and *ς*_1_ with **P**^(0)^, respectively;3: repeat4:  *t* = *t* +1;5:  Apply the IPM to solve the subproblem (P3-1) and obtain the optical power allocation matrix **P**^(*t*)^;6:    repeat7:     $\varsigma_{0}\leftarrow \gamma_{v,m}\left(P^{\left(t\right)}\right)$;8:     Calculate $\vartheta (\varsigma_{0})$ by (35);9:     Calculate $\varepsilon \left(\varsigma_{0}\right)$ by (36);10:   Calculate *ς*_1_ by (41);11:   *m* = *m* + 1;12:   **until**
*m*> *M*13:**until**: ${\left\|\left(P^{\left(t+1\right)}\right)-\left(P^{\left(t\right)}\right)\right\|}_{F}\leq \epsilon_{1}$;14:Output: $P^{*}$.

### 3.4. Optimization of User Association

Given the fixed **B**, **P**, and **D**, the RF achievable data rate *S_m_* is constant. Thus, the problem (P0) can be written as:

(44) $$\begin{array}{c} (P4)\;\underset{A}{\max}\sum_{m=1}^{M}R_{m}\left(A\right)\\ \;s.t.\;(15h),(15i), \end{array}$$

(44a) $$R_{m}\geq R_{\min}-S_{m},\forall m\in M.$$

We begin by relaxing the constraint in (15h) to $0\leq \alpha_{v,m}\leq 1$, which represents the association probability of the *m*-th IUE being served by VLC AP *v*.

Next, based on the definition of the SINR in (4), the objective function *R_m_* (**A**) exhibits a structure similar to that of *R_m_* (**P**), which suggests that the relaxed formulation of (P4) is still a non-convex problem. Hence, the MM algorithm is adopted to address subproblem (P4), and its detailed procedure has already been described in the context of optical power allocation subproblem (P3). Due to the structural similarity between (P3) and (P4), the corresponding property proofs and algorithm descriptions are not reiterated here.

*Remark 2:* The user association variables **A** derived from problem (P4) are relaxed to continuous values in the range [0,1]. Therefore, a reconstruction procedure is required to recover the corresponding binary allocation variables. To reconstruct the binary variables, the objective function of problem (P4) is maximized, and the corresponding optimal indices are then obtained as:

(45) $$v*=\arg\max_{v}\frac{\partial \left(\sum_{m\in M}R_{m}\left(A\right)\right)}{\partial \alpha_{v,m}},$$

(46) $$\;m^{*}=\arg\max_{m}\frac{\partial \left(\sum_{m\in M}R_{m}\left(A\right)\right)}{\partial \alpha_{v,m}},$$

where $\alpha_{v^{*},m}^{*}=1$ and $\alpha_{v,m^{*}}^{*}=1$ represent the suboptimal user allocation variables, respectively.

### 3.5. Proposed BCD-Based AIOA

Building upon the four subproblems described above, we propose an effective BCD-based AIOA to achieve a locally optimal solution for problem (P0). In each iteration, one variable block is updated with the others fixed, and the procedure continues until convergence. The complete alternating iterative optimization procedure is described in Algorithm 2. In each iteration, the starting point for each subproblem is the solution from the previous iteration, which guarantees that a feasible starting solution is always available.

Following that, we provide an analysis of the convergence behavior of the proposed AIOA. Specifically, we prove that the objective function of problem (P0) does not decrease at the *τ*-th iteration, which can be written as:

(47) $$\begin{array}{ll} \Gamma \left(A^{\left(\tau \right)},P^{\left(\tau \right)},B^{\left(\tau \right)},D^{\left(\tau \right)}\right) & \overset{\left(a\right)}{\leq }\Gamma \left(A^{\left(\tau \right)},P^{\left(\tau \right)},B^{\left(\tau \right)},D^{\left(\tau +1\right)}\right)\\ & \overset{\left(b\right)}{\leq }\Gamma \left(A^{\left(\tau \right)},P^{\left(\tau \right)},B^{\left(\tau +1\right)},D^{\left(\tau +1\right)}\right)\\ & \overset{\left(c\right)}{\leq }\Gamma \left(A^{\left(\tau \right)},P^{\left(\tau +1\right)},B^{\left(\tau +1\right)},D^{\left(\tau +1\right)}\right)\\ & \overset{\left(d\right)}{\leq }\Gamma \left(A^{\left(\tau +1\right)},P^{\left(\tau +1\right)},B^{\left(\tau +1\right)},D^{\left(\tau +1\right)}\right). \end{array}$$

where (a) holds since **D**^(*τ* + 1)^ is defined as:

(48) $$D^{\left(\tau +1\right)}=\underset{D}{\arg\max}\left(\Gamma \left(A^{\left(\tau \right)},B^{\left(\tau \right)},P^{\left(\tau \right)},D^{\left(\tau \right)}\right),\Gamma \left(A^{\left(\tau \right)},B^{\left(\tau \right)},P^{\left(\tau \right)},D^{\left(*\right)}\right)\right),$$

where D* denotes the optimal solution of problem (P1-2), and (b) holds because the first-order Taylor expansion at the given points of problem (P2-2) is tight, and its solution is optimal. Since the optimal objective of problem (P2-2) forms a lower bound for problem (P2-1), the overall objective function is guaranteed to be nondecreasing during the iterative process. In the same way, (c) and (d) follow from the optimality conditions of convex optimization, which is similar to (b). Thus, problem (P0) achieves a non-decreasing objective value with every iteration. The proposed AIOA is guaranteed to converge, since the objective function of problem (P0) is upper-bounded by a finite constraint.

We now turn to the analysis of the computational complexity of the proposed AIOA. Specifically, the computational complexity of each subproblem is first analyzed, and the total complexity of our proposed algorithm is then summarized for completeness.

SCA method for subproblem (P1): Applying the IPM to solve problem (P1-2) results in a complexity of Ψ_1_ = 𝒪((*MK*)^3.5^log_2_(1/*μ*_1_)) [31], where *μ*_1_ represents the convergence threshold of the IPM.

Relaxed iterative method for subproblem (P2): Similarly, applying the IPM to solve the convex problem (P2-2) yields a computational complexity of Ψ_2_ = 𝒪((*MK*)^3.5^log(1/*μ*_2_)), where *μ*_2_ represents the convergence threshold of the IPM.

MM algorithm for subproblems (P3) and (P4): The overall complexity of Algorithm 1 is mainly determined by iteratively solving (P3-1) and the update of constant parameters. Assuming the convergence threshold of the IPM is *μ*_3_, solving (P3-1) once incurs a complexity of 𝒪((*M + V*)^3.5^log(1/*μ*_3_)). The algorithm converges in about 𝒪(log(1/*ϵ*_1_)) [41] iterations. Furthermore, the computational cost of the variable update step is 4*M*. For problem (P4), an additional computational complexity of 𝒪(*MV*) is required to enforce discreteness on the relaxed solution. Since the computational load of this process is a lower-order linear term compared with that of the main optimization based on the IPM, it can therefore be neglected. Hence, the total computational complexity of Algorithm 1 is expressed as Ψ_3_ = 𝒪((4*M* + (*M + V*)^3.5^) log(1/*μ*_3_) log(1/*ϵ*_1_)).

Since Algorithm 2 alternately solves the subproblems (P1), (P2), (P3) and (P4), its number of iterations is approximately 𝒪(log(1/*ϵ*)) [41]. Therefore, the total computational complexity of the AIOA in solving problem (P0) is Ψ = 𝒪((Ψ_1_ + Ψ_2 +_ Ψ_3_) log(1/*ϵ*)).
**Algorithm 2.** BCD-Based Alternating Iterative Optimization Algorithm (AIOA)1:**Input:** $\left\{h_{v,m}\},\{h_{b,m}\},\{h_{m,k}\},\{h_{k}\},\{h_{b,k}\right\}$, and the convergence threshold *ϵ* > 0.2:**Initialization**: $\tau =0,P^{\left(0\right)},D^{\left(0\right)},A^{\left(0\right)}$, and $B^{\left(0\right)}$ are initialized.3:repeat:4:Solve problem (P1-2) for the given $\left\{A^{\left(\tau \right)},B^{\left(\tau \right)},P^{\left(\tau \right)}\right\}$, and obtain the optimal RF power allocation matrix $\left\{D^{\left(\tau +1\right)}\right\}$;5:Solve problem (P2-2) for the given $\left\{A^{\left(\tau \right)},P^{\left(\tau \right)},D^{\left(\tau +1\right)}\right\}$, and obtain the optimal RF spectrum reuse allocation matrix $\left\{B^{\left(\tau +1\right)}\right\}$;6:Given $\left\{A^{\left(\tau \right)},B^{\left(\tau +1\right)},D^{\left(\tau +1\right)}\right\}$, solve problem (P3-1) by the MM algorithm and obtain optical power allocation matrix $\left\{P^{\left(\tau +1\right)}\right\}$;7:Given $\left\{P^{\left(\tau +1\right)},D^{\left(\tau +1\right)},B^{\left(\tau +1\right)}\right\}$, solve problem (P4) by the heuristic MM algorithm and obtain the optimal user association matrix $\left\{A^{\left(\tau +1\right)}\right\}$;8:*τ* = *τ* + 1;9:**until**
$\left|\Gamma^{\left(\tau \right)}-\Gamma^{\left(\tau -1\right)}\right|<\epsilon$;10:**Output:** $\left\{A^{\left(\tau +1\right)},B^{\left(\tau +1\right)},P^{\left(\tau +1\right)},D^{\left(\tau +1\right)}\right\}$.

## 4. Numerical Results and Analysis

In this section, we present the simulation results for the aggregated VLC–RF vehicular networks. We first evaluate the convergence behavior of the proposed AIOA and then compare the performance of the algorithms for subproblems (P1), (P2), (P3), and (P4) with that of several baseline schemes. In addition, we also analyze how key parameters affect the performance of the network.

### 4.1. System Parameters

The simulation setup for the freeway scenario is based on a two-lane highway, where a single RF AP located at the center of the simulation area provides coverage for all vehicles, and five VLC APs are uniformly deployed along the road with an inter-spacing of 20 m [31]. Both the lane width and average inter-vehicle distance were taken from [34]. Vehicles follow a spatial Poisson process for random distribution along the road, with the vehicle density being dependent on their speed *c_v_*. From the generated vehicles, *M* V2I links are chosen at random, while *K* V2V links are established between each vehicle and its nearest neighboring node. Additionally, Monte Carlo simulations are employed to evaluate the performance of our proposed algorithm. For each realization, a uniformly distributed random value in the range [0,1] is generated. If its value is below *ρ*, indicating LoS availability, the LoS component is assumed to be present; otherwise, it is treated as absent [45]. The simulation parameters are listed in Table 2, unless otherwise specified [31].

### 4.2. Baseline Schemes

To highlight the advantages of resource allocation optimization, the proposed AIOA is compared with the following benchmark schemes.

(1) **Ideal scheme**: The resource allocation strategy is designed based on the instantaneous CSI for all vehicular links, which constitutes an idealized assumption in practice.
(2) **Random RF spectrum reuse**: The spectrum occupied by the IUEs is randomly reused by the VUE pairs, meaning that the spectrum reuse variables **X** are not subject to optimization within the AIOA.
(3) **Average optical power allocation**: In this case, each VLC AP distributes its transmit power equally among all IUEs. The matrices **D**, **X**, and **A** are obtained by the proposed AIOA.
(4) **SCG scheme** [20]: This method is applied to assign the user association coefficient A according to the strongest channel gain (SCG) [20], while the matrices D, B, and P are optimized by the proposed AIOA.

### 4.3. Simulation Analysis of the Proposed Algorithm

**Figure 2:**
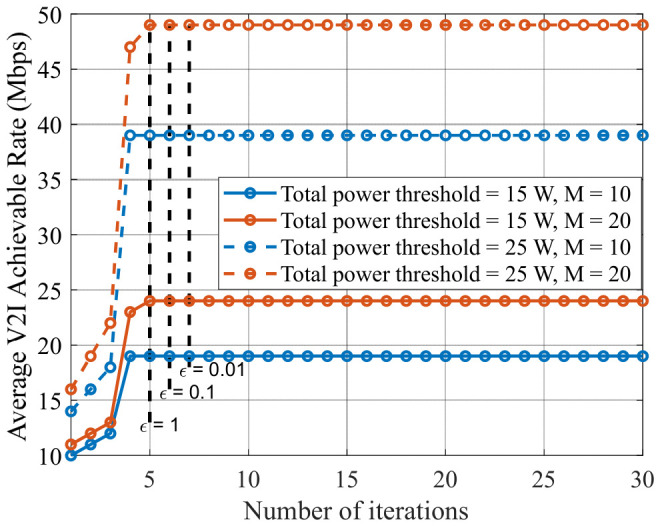
Convergence behavior of the proposed AIOA for different total power thresholds and number of IUEs.

Figure 2 illustrates the convergence behavior of the proposed AIOA for different total power thresholds and number of IUEs. The black lines indicate the number of iterations required for convergence under different threshold values of *ϵ*. According to the simulation results, the algorithm is observed to converge within approximately seven iterations, as the iterative difference *ϵ* at this stage has already fallen below 0.01 Mbps. In addition, the average V2I achievable rate does not decrease during the iterations, confirming the conclusion in Section 3.5 and demonstrating that the proposed algorithm has acceptable computational complexity. Moreover, by comparing the converged average V2I achievable rate values, it can be clearly observed that the proposed algorithm achieves a remarkable improvement over the initial state as the total power threshold *U_T_* increases, which indicates improvements in the SINRs of both the RF AP and the VLC APs, respectively. As an example, for *M* = 20, the proposed algorithm outperformed the initial state by approximately 14 Mbps and 32 Mbps at total power thresholds of 15 W and 25 W, respectively. Moreover, the improvement in the average V2I achievable rate resulting from an increase in the number of IUEs becomes more significant in the high-SINR region of the aggregated VLC–RF networks.

**Figure 3:**
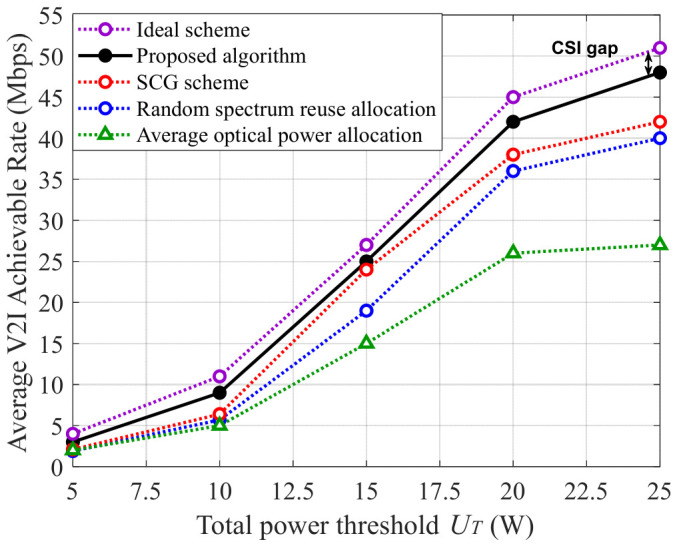
Average V2I achievable rate versus the total power threshold for different benchmark schemes.

Figure 3 presents an analysis of the average V2I achievable rate versus the total power threshold *U*_T_ (W) for different benchmark schemes. The influence of different variables on the average V2I achievable rate optimization is illustrated through comparisons between the proposed algorithm and the baseline schemes. Simulation results indicate that the average optical power allocation scheme performed worse than all other methods, which demonstrates that optical power allocation impacts the V2I achievable rate more significantly than user association and RF spectrum reuse allocation. Furthermore, when the total power threshold was 25 W, the proposed scheme outperformed the SCG scheme and the random spectrum reuse configuration by about 6 Mbps and 8 Mbps, respectively. This result highlights the importance of user association optimization and the necessity of a careful design of spectrum reuse. Moreover, it was observed that the growth of the average V2I achievable rate of all algorithms first increased and then gradually converged as the total power threshold rose. This is because when the total power threshold becomes relatively large, interference from other VLC APs and VUE pairs becomes the main source of disturbance. Consequently, higher transmit power at the VLC APs and the RF AP leads to stronger interference, making it difficult to improve the average V2I achievable rate. Finally, the ideal scheme utilizing real-time CSI achieved the best performance, followed closely by the proposed algorithm with a small performance gap.

### 4.4. Simulation Analysis of Key Parameters

**Figure 4:**
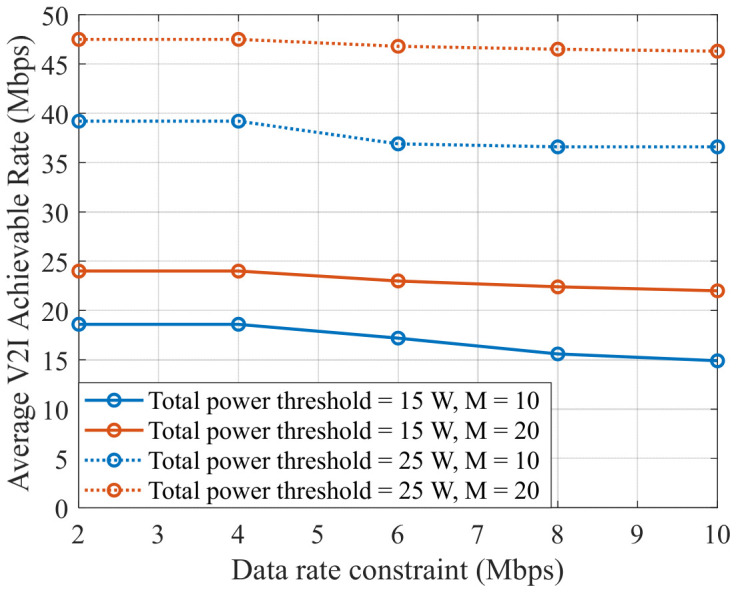
Average V2I achievable rate versus the data rate constraint for different total power thresholds and number of IUEs.

Figure 4 illustrates the average V2I achievable rate versus the data rate constraint *R*_min_ for different total power thresholds and number of IUEs. As shown in Figure 4, when *R*_min_ increased, the average V2I achievable rate of the proposed AIOA first remained stable and then gradually decreased. When *R*_min_ was lower than the achievable rate of each IUE, the rate constraint became inactive, resulting in a constant average V2I achievable rate of the aggregated vehicular network. As *R*_min_ continued to increase, some IUEs failed to satisfy the data rate constraint. Consequently, the centralized controller needed to reallocate additional resources from other IUEs to these IUEs to ensure the feasibility of the rate constraint, which in turn led to a lower average V2I achievable rate. However, it is evident that the decline in the average V2I achievable rate for *M* = 20 was significantly slower than that for *M* = 10, indicating that increasing the number of IUEs enables more IUEs to meet the QoS requirement and reduces the impact of the data rate constraint.

**Figure 5:**
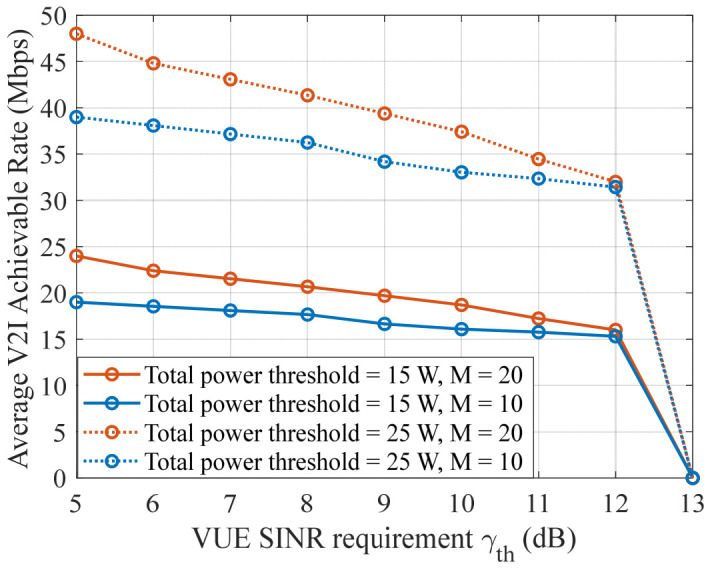
Average V2I achievable rate versus the VUE SINR requirement for different total power thresholds and number of IUEs.

Figure 5 compares the average V2I achievable rate versus the VUE SINR requirement *γ*_th_ for different total power thresholds and number of IUEs. It was observed that the average V2I achievable rate decreased with an increase in the minimum VUE SINR requirement *γ*_th_. This is because increasing the VUE SINR requirement compels the system to allocate higher transmit power to the VUE pairs in order to satisfy the VUE SINR constraint. However, a higher VUE transmit power leads to increased interference to the IUEs, which in turn decreases the average V2I achievable rate. When *γ*_th_ ≥ 12 dB, the average V2I achievable rate fell to zero. This occurred because the system is unable to further increase the VUE transmit power due to the maximum VUE power constraint. As a result, the inability to meet the minimum VUE SINR requirement leads to the infeasibility of the resource allocation problem.

**Figure 6:**
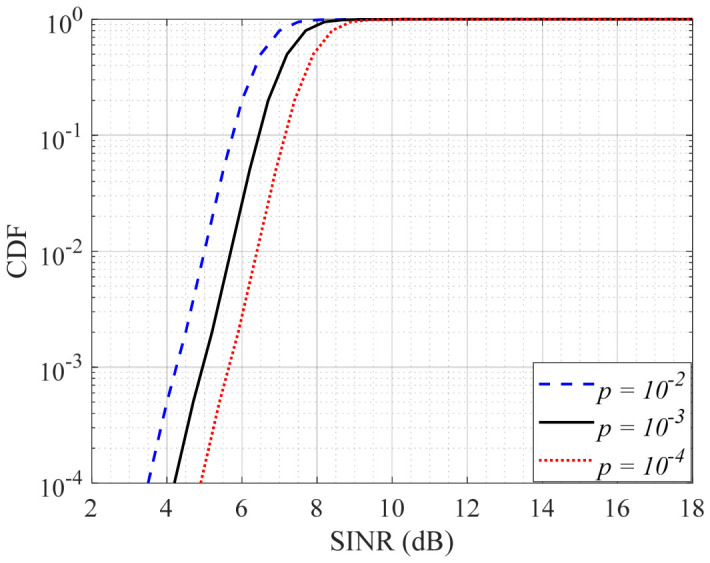
CDF of the SINR for an arbitrary VUE pair with T = 1 ms under different target outage probabilities *p*.

Figure 6 illustrates the cumulative distribution function (CDF) of the received SINR for an arbitrary VUE pair under different target outage probabilities *p*, where the SINR threshold is set to 5 dB. As shown in the figure, the reliability constraint based on the VUE SINR requirement is well-satisfied, which verifies the effectiveness of the proposed AIOA.

**Figure 7:**
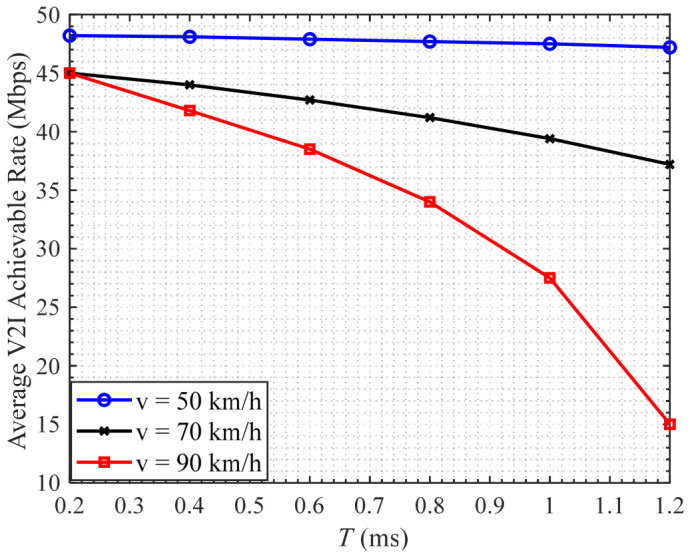
Average V2I achievable rate with varying feedback period T.

Figure 7 demonstrates the average V2I achievable rate with an increasing CSI feedback period that indicates the channel latency. From the figure, the average V2I achievable rate decreases as the reporting period *T* grows. This is because growing *T* increases the uncertainty of V2V channels at the RF AP, motivating the RF AP to act conservatively when controlling the IUEs’ transmit powers to meet the reliability constraint of V2V links, which suffer from interference generated by IUEs. As the vehicle speed increases from 50 to 90 km/h, the average V2I achievable rate drops, since higher speed induces a larger Doppler shift, which also increases channel uncertainty at the RF AP. Another reason for such degradation is due to sparser traffic according to the simulation setup, which on average, increases inter-vehicle distance and gives rise to less reliable V2V links with lower received power. As such, less interference from IUEs can be tolerated given the maximum transmit power constraints of VUEs, leading to less power being allocated to IUEs and decreasing their achievable rate. It is also interesting to note from Figure 7 that the IUE’s average V2I achievable rate was more sensitive to feedback period with a larger vehicle speed.

**Figure 8:**
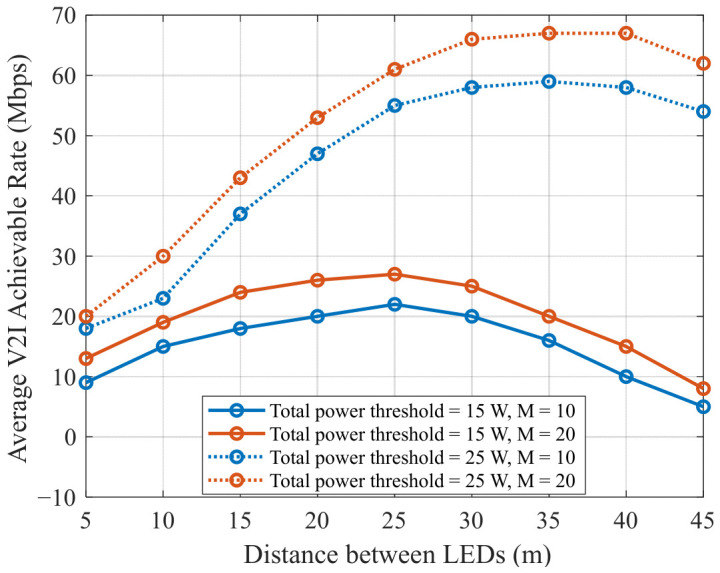
Average V2I achievable rate versus the distance between VLC APs for different total power thresholds and number of IUEs.

Figure 8 illustrates the impact of the distance between VLC APs on the average V2I achievable rate for different total power thresholds and number of IUEs. It can be observed that all four curves exhibited a trend of initial increase followed by a decrease. From the physical-layer perspective, the average V2I achievable rate is mainly determined by the received SINR, which depends on both the desired received optical power and the inter-cell interference. When the VLC AP spacing is small, the coverage areas of adjacent APs highly overlap, leading to strong inter-cell interference and a reduced SINR. As the AP spacing increases, the interference from neighboring APs is weakened, and the SINR is improved, resulting in an increase in the average V2I achievable rate. However, when the AP spacing becomes excessively large, some IUEs are located far from their serving APs or near the cell edge. In this case, the propagation distance and incidence angle increase, which reduces the VLC channel gain and the received optical power. Consequently, the degradation of the desired signal becomes dominant, leading to a decrease in SINR and hence a reduction in the achievable rate. Furthermore, it should be noted that when the total power threshold is high, the spacing between the VLC APs should be appropriately increased, as inter-cell interference becomes the primary factor affecting network performance. In addition, VLC AP spacing should be properly designed for outdoor illumination, as excessive separation between VLC APs can cause illumination non-uniformity and insufficient brightness. Consequently, the selection of VLC AP spacing must account for various parameters, such as lighting quality and communication demands, and should be refined based on realistic deployment environments in future research.

**Figure 9:**
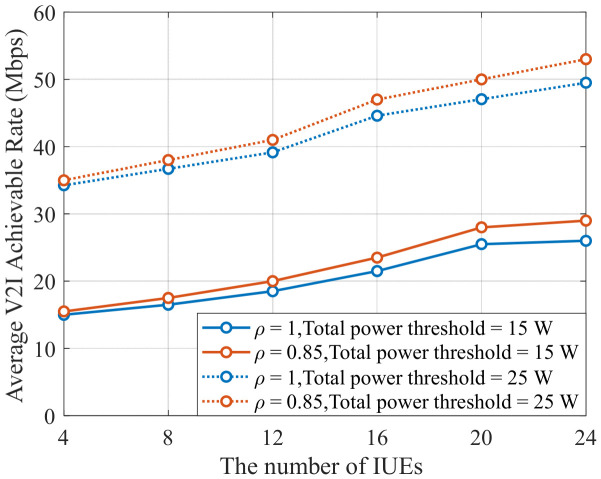
Average V2I achievable rate versus the number of IUEs for different total power thresholds under different *ρ* values.

Figure 9 shows the average V2I achievable rate versus the number of IUEs for different total power thresholds under different *ρ* values. Here, *ρ* = 1 indicates that no blockages occur, whereas *ρ* = 0.85 implies a 15% probability of a blockage existing between the VLC APs and the IUEs. The results show that for different values of *ρ*, increasing the total power threshold leads to a higher V2I achievable rate. Furthermore, when *ρ* = 1, the average V2I achievable rate is not optimal. This is because the strong direct signal effect resulting from a high LoS probability enhances the interference between IUEs and limits further improvement of the average V2I achievable rate. When the blockage probability reaches 15%, the average V2I achievable rate improves, suggesting that a certain level of NLoS propagation can reduce interference among IUEs and improve the average V2I achievable rate.

**Figure 10:**
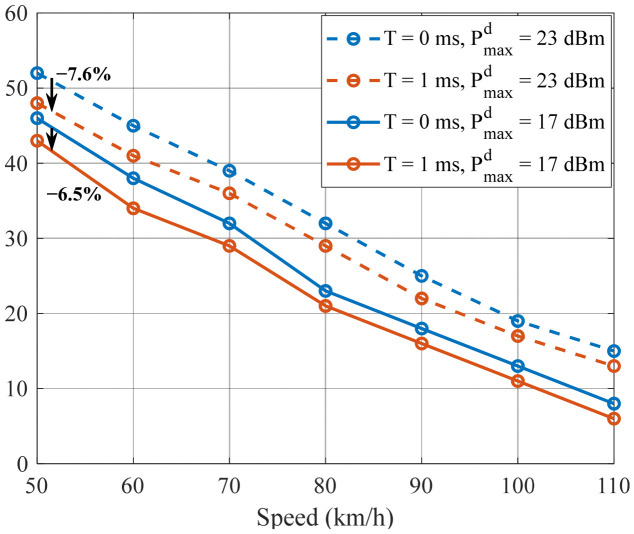
Average V2I achievable rate versus the vehicle speed for different feedback period *T* under different $P_{max}^{d}$ values.

In Figure 10, we analyze the relationship between the average V2I achievable rate versus the vehicle speed for different feedback periods *T* under different $P_{max}^{d}$ values. It is evident from the figure that the average V2I achievable rate decreases as the feedback period *T* increases. Specifically, at a speed of 50 km/h, increasing the feedback period *T* from 0 ms to 0.2 ms resulted in reductions of 7.6% and 6.5% in the average V2I achievable rate under $P_{max}^{d}$ = 23 dBm and $P_{max}^{d}$ = 17 dBm, respectively. In addition, when the feedback period *T* is fixed, reducing the $P_{max}^{d}$ by 6 dBm resulted in a corresponding decrease in the average V2I achievable rate. This is because a larger feedback period *T* increases the uncertainty of the V2V channels at the APs, leading the APs to adopt a conservative power control policy for the IUEs to guarantee the reliability of VUEs that are subject to interference from the IUE transmissions. Moreover, increasing the vehicle speed from 50 to 110 km/h led to a degradation in the average V2I achievable rate based on the simulation setup. Higher vehicle speeds correspond to sparser traffic conditions, yielding larger average inter-vehicle distances and reducing V2V link reliability due to lower received signal power. To ensure reliable V2V connectivity under these conditions, the VUE transmitter must increase its transmit power to offset the increased path loss caused by larger inter-vehicle distances, which in turn generates stronger interference to the IUEs and reduces their achievable rate.

**Figure 11:**
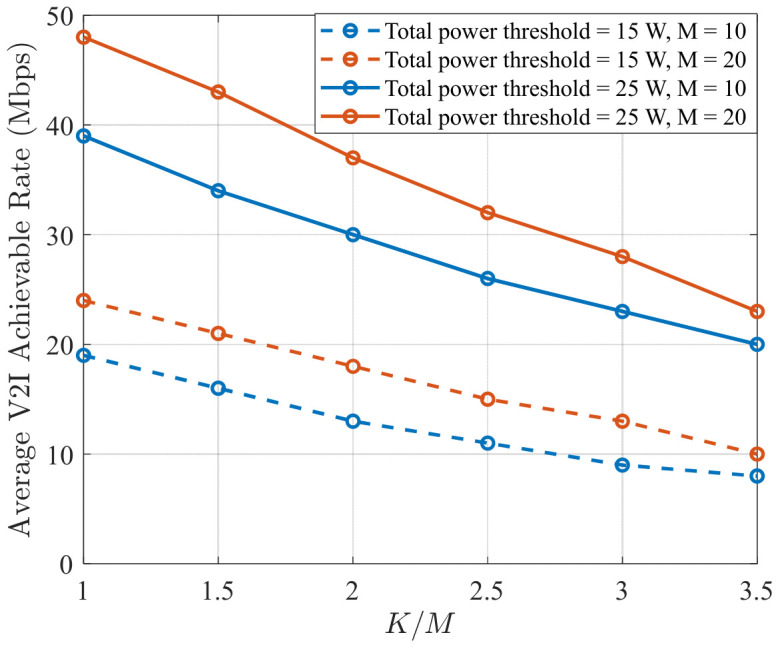
Average V2I achievable rate versus varying number of VUE pairs for different total power thresholds and number of IUEs.

To evaluate the influence of the RF spectrum reuse scheme on the average V2I achievable rate performance in the aggregated VLC–RF vehicular networks, the constraint $\sum_{m\in M}b_{m,k}\leq 1,\;\forall k$ in (19k) was neglected. Consequently, the spectrum assigned to each IUE may be reused concurrently by several VUE pairs. As presented in Figure 11, increasing the number of VUE pairs participating in spectrum reuse led to a reduction in the average V2I achievable rate across all schemes. The primary causes of the average V2I achievable rate degradation are as follows. Each IUE must concurrently share its RF spectrum with a larger number of active VUE pairs. To maintain the reliability of these VUE pairs, the IUE transmit power must be regulated to limit the interference imposed on them. The reduced signal power of each IUE leads to a decline in the average V2I achievable rate. Additionally, a larger count of VUE pairs introduces additional interference to the IUEs, thereby decreasing γ*_m_*. Moreover, increasing the total power threshold from 15 W to 25 W leads to a corresponding improvement in the average V2I achievable rate. When the condition *K*/*M* > 3 holds, there is only a marginal improvement in the average V2I achievable rate performance. These results indicate that the IUEs are heavily impacted by interference from the VUE pairs in this scenario, which is the principal cause of the reduction in the average V2I achievable rate.

## 5. Conclusions

In this work, we investigated aggregated VLC–RF vehicular networks to ensure QoS for V2X communications and enhance ubiquitous coverage. We also considered the channel uncertainty caused by delayed CSI feedback in high-mobility vehicular scenarios. Based on the proposed spectrum reuse model, we formulated an optimization problem that aims to maximize the sum V2I achievable rate while satisfying practical QoS requirements. To solve this problem, we developed a BCD-based alternating optimization algorithm to iteratively update the resource management variables. Specifically, the SCA method was adopted for RF transmit power allocation, a relaxed iterative method was used for RF spectrum reuse allocation among VUE pairs, and the MM algorithm was applied to optical power allocation and user association. The proposed algorithm was shown to converge to a stable solution. Numerical results demonstrated that the proposed algorithm provided a computationally feasible solution for improving the sum V2I achievable rate and outperformed the considered baseline schemes, especially under high SINR requirements. The results also indicate that key system parameters, such as the total power threshold, VLC AP spacing, and QoS requirements of different vehicles, should be carefully configured in aggregated VLC–RF vehicular networks. In summary, the studied aggregated VLC–RF vehicular network and the proposed algorithm can efficiently and jointly utilize VLC and RF AP resources to improve the sum V2I achievable rate while providing ubiquitous connectivity for vehicles.

In future work, we will investigate the extension of the proposed framework to support multiple VLC AP associations for IUEs. In the current work, each IUE was assumed to be associated with at most one VLC AP in order to reduce the complexity of AP coordination and interference management. The potential benefits of multiple VLC AP associations, as well as the possible performance degradation caused by additional interference and signaling overhead, will be rigorously analyzed in our future study. In addition, we will further extend the proposed framework by considering more realistic V2V mobility characteristics, including relative vehicle speed, time-varying inter-vehicle distance, link duration, and V2V reliability performance. These extensions will help evaluate the proposed resource allocation scheme more comprehensively in real vehicular environments.

## Figures and Tables

**Table 1:** X vehicular networks.

Refs.	System Model	Contribution
[23]	An RF-based vehicular network with relay-assisted multiple-user communication.	**Design aspect:** Cross-layer power allocation and relay selection are investigated. **Objective:** Network sum-rate maximization. **Technique:** A multi-tier water-filling algorithm is used for power allocation, and a pricing-based mechanism is adopted for relay selection.
[24]	An RF-based V2X network consisting of IUEs, safety-related VUEs, and non-safety VUEs.	**Design aspect**: Resource allocation among different types of V2X users is investigated. **Objective:** Network sum-rate maximization. **Technique:** The latency and reliability requirements of safety-critical V2X are transformed into constraints based on slowly varying CSI. An optimization problem supporting non-orthogonal V-UE resource sharing is formulated, and a three-stage resource block sharing and power allocation algorithm based on clustering and matching theory is proposed for efficient solution.
[25]	An RF-based V2X network consisting of V2I and V2V links.	**Design aspect**: Spectrum sharing and power allocation problem. **Objective:** Maximize the system capacity including all V2I links and V2V links. **Technique:** The joint problem is separated into two sub-problems: channel allocation and power control. The base station centrally performs channel allocation based on graph matching, while vehicle users conduct distributed power control using a deep deterministic policy gradient algorithm.
[26]	An RF-based vehicular network with IUEs and VUE pairs.	**Design aspect**: Spectrum reuse and power allocation are considered. **Objective:** The overall energy efficiency while satisfying the SINR requirements of VUEs. **Technique:** An optimization framework is proposed for power splitting adjustment and power allocation. In this framework, the power allocation problem is addressed based on the Lagrangian dual function, while the power splitting adjustment problem is solved using Dinkelbach’s method.
[27]	An RF-based V2V vehicular network.	**Design aspect**: Frequency bandwidth, power allocation, and modulation/coding of V2V communications is considered. **Objective:** Maximization of the minimum SINR of CUEs. **Technique:** Lagrange dual decomposition and stochastic network analysis are used to solve the formulated problem.
[28]	An V2X network with multiple RF APs and multiple cars.	**Design aspect**: Power allocation and vehicle-network association. **Objective:** Maximize the overall QoE utility of V2X vehicles. **Technique:** An optimization framework is proposed for vehicle–network association and power allocation. In this framework, the vehicle–network association problem is addressed based on greedy algorithm, while the power allocation problem is solved using penalty function algorithm.
[29]	A time-varying RF vehicular network with an RF AP is considered, where both large-scale and small-scale fading are taken into account.	**Design aspect**: Spectrum reuse and power allocation are considered. **Objective:** Maximize the overall V2I link throughput. **Technique:** The joint problem is separated into two sub-problems, spectrum reuse and power control. The power control problem is addressed closed-form optimal power control, while the spectrum reuse problem is solved using Hungarian matching algorithm.
[30]	An RF-based vehicular network with delayed CSI feedback.	**Design aspect**: Spectrum reuse and power allocation are considered. **Objective:** Maximize the overall V2I link throughput. **Technique:** The whole algorithm can be divided into two stages, first, the optimal power is analytically derived, and then graph matching is used to obtain the optimal spectrum matching.
[19]	An aggregated VLC–RF system with one RF AP, one VLC AP and IUEs.	**Design aspect**: Time resource allocation and power allocation. **Objective:** Maximize the lower bound of energy efficiency within one period. **Technique:** An alternating iterative optimization method is adopted to solve the time–resource allocation and power allocation subproblems.
[31]	An IRS-assisted aggregated VLC–RF vehicular system with multiple VLC APs, one RF AP and IUEs.	**Design aspect**: VLC optical subchannel assignment, VLC power allocation, RF power allocation, and RF intelligent reflecting surface (IRS) phase-shift configuration and optical IRS unit arrangement. **Objective:** Maximize the energy efficiency. **Technique:** With the other variables fixed, gradient descent is used to optimize the RF IRS phase shift; Lagrangian relaxation heuristics is used to optimize the optical subchannel assignment; minorization–maximization is used to optimize the OIRS unit arrangement; Dinkelbach-based sequential fractional programming is used to jointly optimize the VLC and RF power allocation matrices.

**Table 2:** Simulation parameters.

	Parameter	Value
VLC	Refractive index, *χ*	1.5
	LED semi-angle at half-power *φ*_1/2_	60°
	PD active detection area, *ε*	1 cm^2^
	FOV of a photodetector, *ξ*_FOV_	70°
	Lambertian order, Ω	1
	Dimming level, *τ*	0.6
	Gain of optical filter, *T_S_*(*ξ*)	1
	the noise power of the VLC link, $\sigma_{VLC}^{2}$	−96.98 dBm
	Responsivity of the PD, *ϖ*	0.5 A/W
	Bandwidth of VLC links, *Q*_1_	10 MHz
	Maximum power of each VLC AP, *P_v_*_,max_	4 W
	Static power consumption of a VLC AP,*U*_1_	1W
	Number of VLC APs, *V*	5
RF	Carrier frequency, *f*_c_	2 GHz
	Maximum power of the RF AP, *W*_max_	4 W
	Path loss decay exponent for CUEs, *β*	3
	Path loss decay exponent for VUE pairs, *β*	2.2
	Maximum transmit power of VUE pair, $P_{max}^{d}$	23 dBm
	the noise power of the VLC link, $\sigma_{RF}^{2}$	−94.20 dBm
	Bandwidth of RF links, *Q*_2_	5 MHz
	Pathloss constant for CUEs, κ	128.1 + 37.6 log(d[km])
	Pathloss constant for VUE pairs, κ	148.1 + 40 log(d[km])
	Minimum SINR threshold for VUE pair, γ_th_	5 dB
	Static power consumption of the RF AP, *U*_2_	2 W
	Shadow fading standard deviation (V2I), ν	8 dB
	Shadow fading standard deviation (V2V), ν	3 dB
	Fast fading, *η*	Rayleigh model (*η* = 3dB)
System	Data rate constraint, *R*_min_	4 Mbps
	Total power threshold, *U*_T_	25 W
	Number of IUE_S_ and VUE pair (*M*, *K*)	*M* = *K* = 10
	Feedback period, *T*	1 ms
	Average inter-vehicle distance	2.5*c_v_*

## Data Availability

The original contributions presented in this study are included in the article. Further inquiries can be directed to the corresponding author.
